# First person – Kathrin Pfeifer

**DOI:** 10.1242/dmm.049751

**Published:** 2022-08-16

## Abstract

First Person is a series of interviews with the first authors of a selection of papers published in Disease Models & Mechanisms, helping early-career researchers promote themselves alongside their papers. Kathrin Pfeifer is first author on ‘ Patient-associated mutations in *Drosophila* Alk perturb neuronal differentiation and promote survival’, published in DMM. Kathrin is a postdoc in the lab of Ruth Palmer at the University of Gothenburg, Gothenburg, Sweden, investigating aberrant Alk signalling in *Drosophila*.


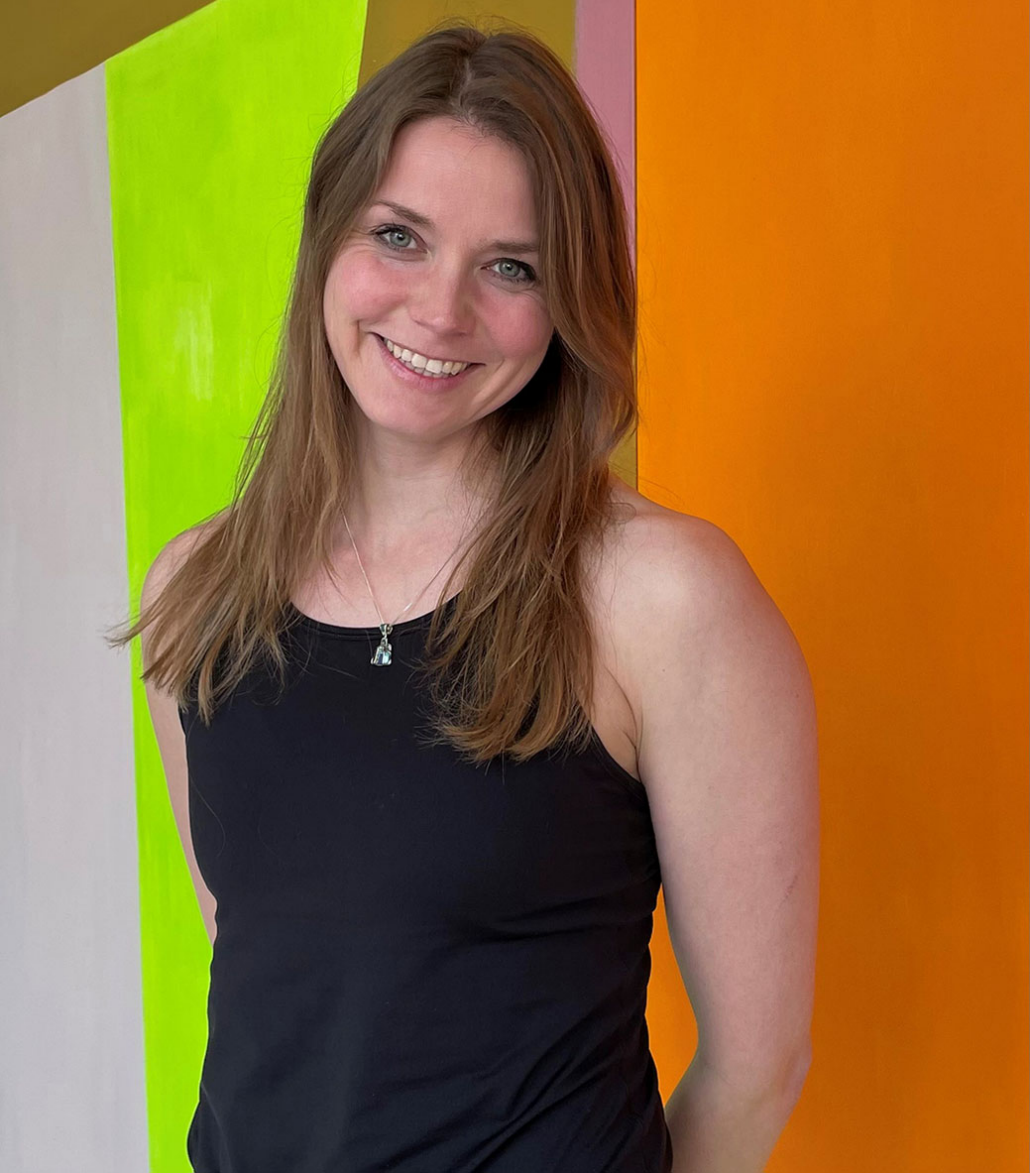


**Kathrin Pfeifer**

**How would you explain the main findings of your paper to non-scientific family and friends?**

Highly aggressive neuroblastoma in children can be associated with mutations in ALK. These mutations correlate with aggressive tumor formation in patients. Neuroblastomas that are ALK positive respond to treatment with ALK tyrosine kinase inhibitors, but combinations with other therapeutic agents will likely be required to maximise their effect. We use the easy to maintain, analyse and manipulate fruit fly as a highly controllable genetic model in order to understand the effects of ALK mutation at the cellular level. I created disease-relevant point mutations in Alk, and we found that these mutations resulted in a decrease in apoptosis in Alk-expressing tissues of the central nervous system (CNS). We also found that aberrant signalling leads to ectopic phosphorylation of ERK during embryo development and a cell fate change in the mushroom body lineage of the fly CNS, which is maintained until adulthood.

**What are the potential implications of these results for your field of research?**

It's important to note that the Alk mutations created here in *Drosophila* are still ligand dependent, which might be important for neuroblastoma. If we can understand better how the mutated receptor leads to aberrant signalling, it might help to find new therapeutics. Also, the cell fate changes together with the decreased apoptosis that occurs through Alk signalling could reflect a baseline differentiation defect that underpins tumor development.

**What are the main advantages and drawbacks of the model system you have used as it relates to the disease you are investigating?**

The advantages with the fruit fly are primarily that it is an invertebrate model that is easy to handle and maintain, and we have great tools to genetically manipulate with CRISPR/Cas9 in clean genetic backgrounds. We can keep lethal mutations over so-called balancer chromosomes, and we have overexpression tools that make it easy to study tissue-specific overexpression. Drawbacks include that it's a bit harder to get funding because most funders value more vertebrate models or human cell culture.

“It was very surprising to find that the ALK gain-of-function mutations described in human are still ligand dependent in *Drosophila* at endogenous expression levels.”

**Figure:**
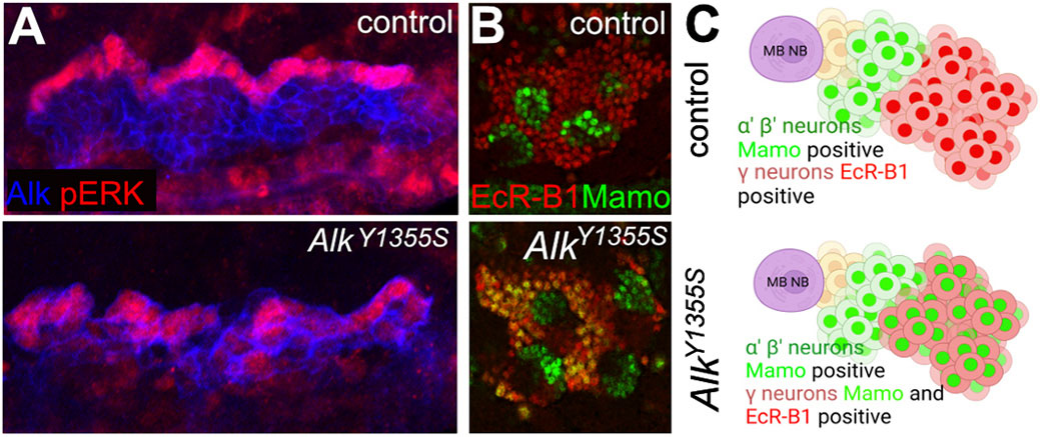
**Analysis of aberrant Alk signalling in the embryonic visceral mesoderm and mushroom body lineage of larval brains of *Alk^Y1355S^ Drosophila* mutants.** (A) Ectopic phospho-ERK (pERK; red) in the visceral mesoderm (blue) of *Drosophila* embryos in stage 10/11 in the mutant *Alk^Y1355S^* compared to the control. (B,C) In the γ-lineage (red) of the mushroom body lineage, aberrant *Alk^Y1355S^* signalling leads to precocious Mamo (green) expression in wandering stage 3 larval brains of the mutants.

**What has surprised you the most while conducting your research?**

It was very surprising to find that the ALK gain-of-function mutations described in human are still ligand dependent in *Drosophila* at endogenous expression levels, as we were not expecting this. I was also surprised to see that Raf–Ras–MAPK signalling output can vary a lot, which might also be caused by access to the ligand or issues in degradation/recycling. Degradation and recycling defects have been reported in gain-of-function mutations in other receptor tyrosine kinases that lead to increased and prolonged signalling. I think it's surprising that we haven't studied ALK mutations in more detail at a subcellular level yet. Finally, I was surprised and happy that we could finally identify a molecular readout for changed Alk signalling in the mushroom body lineage of the Alk mutant brains. Aberrant Alk signalling changes transcriptional readouts, which we could nicely show by staining for the transcription factor Mamo, which is precociously expressed in Alk mutants.

**Describe what you think is the most significant challenge impacting your research at this time and how will this be addressed over the next 10 years?**

It is challenging to translate what we see in *Drosophila* over to humans when it comes to Alk mutations. There is a great conservation between the species but still there are differences that raise questions. For example, is the dependency on the ligand we observe in *Drosophila* something that is occurring in human too? *In vitro* experiments suggest they are ligand independent; however, we need to consider that levels of expression may mean that ligand dependency has been missed. It makes me want to eliminate the Alk ligands in neuroblastoma cell culture to study the real impact of the ligands in a setting without overexpression but genetic modification. Generally, the ALK mutations that are frequently found in neuroblastoma patients should be studied so that we understand the effect on a subcellular level. Is it aberrant signalling due to auto-activation? Is it a degradation failure that leads to prolonged signalling? Is it impaired recycling? Is the recycling pace something that impacts how many cells will get access to the ligand? Or other cellular changes that might lead to prolonged signalling, which changes the cell transcription and the fate? I think it is important to fully understand the dynamics of how mutated receptors behave. The cell fate changes we observe in *Drosophila*, with precocious expression of Mamo induced by aberrant Alk signalling, is also something that should be addressed further in human neuroblastoma cell lines and models.

**What changes do you think could improve the professional lives of early-career scientists?**

There is no standard for students, PhD students and postdocs when it comes to education and trainee programs. Depending on where the young scientist is employed, the possibilities to participate in trainee programs are limited or non-existing. In order to maximise the outcome for early-career scientists, I would propose standard training programs to learn and improve scientific writing for publications, grant applications and to improve the English skills for those that are not native speakers. Especially for students and PhD students, it would be beneficial to have more education and training in reading scientific articles and how to evaluate the own project progress. Regular practical courses to acquire new wet lab skills and techniques that can be implemented into the own research can help the young scientist to develop. And of course, increased funding opportunities for researchers working on invertebrate model organisms.

**What's next for you?**

I will focus on revealing why the *Alk^Y1355S^* mutation, and other Alk mutants found in neuroblastoma, are behaving as they do. I aim to use BioID labeling approaches to explore this further, along with high-resolution microscopy to track mutated Alk subcellularly.
